# Anterior segment characteristics in normal and keratoconus eyes evaluated with a new type of swept-source optical coherence tomography

**DOI:** 10.1371/journal.pone.0274071

**Published:** 2022-09-01

**Authors:** Kook Young Kim, Seongjun Lee, Young Joon Jeon, Ji Sang Min

**Affiliations:** 1 Nuri Eye Hospital, Deajeon, Korea; 2 Kim’s Eye Hospital, Seoul, Korea; Save Sight Institute, AUSTRALIA

## Abstract

**Purpose:**

This study aimed to evaluate and compare the discriminating ability of corneal elevation maps generated using a swept-source optical coherence tomography (SS-OCT) (SS-OCT ANTERION, Heidelberg Engineering, Heidelberg, Germany), which was estimated with different reference surfaces, to distinguish normal corneas from those with keratoconus and keratoconus suspect.

**Methods:**

A total of 126 eyes of patients, which comprised 43, 37, and 46 keratoconus, keratoconus suspects, and normal controls, respectively, were included in this study. The anterior and posterior elevations at the thinnest point under the best-fit sphere (BFS) and toric-ellipsoid (BFT), respectively, and other corneal parameters were measured using the SS-OCT. In addition, the receiver operating characteristic (ROC) curve analysis and cut-off value were calculated to evaluate the diagnostic ability of the corneal elevation values in differentiating keratoconus and keratoconus suspects from normal eyes.

**Results:**

The mean total keratometric and corneal elevation values were significantly higher in the keratoconus group than in the other groups. Pachymetric parameters exhibited the lowest values for keratoconus. In addition, ROC curve analyses showed a high accuracy of the thinnest point anterior and posterior BFT for both keratoconus and keratoconus suspects and normal controls (area under the ROC were 0.969 and 0.961, respectively). Furthermore, the optimal cut-off point of the posterior elevation at the thinnest point under BFT was 16.44 μm (sensitivity and specificity of 86% and 98%, respectively) for differentiating keratoconus from normal and keratoconus suspect eyes.

**Conclusions:**

The elevation map using the BFS and BFT references measured with the anterior segment SS-OCT is considered an effective indicator for keratoconus diagnosis. Therefore, the anterior segment SS-OCT can effectively differentiate keratoconus from suspected keratoconus and normal corneas by measuring parameters such as posterior and anterior elevations, pachymetry, and keratometry.

## Introduction

Keratoconus is an asymmetric, progressive disorder that is characterized by the thinning and weakening of the stromal layer. Consequently, it changes the anterior and posterior corneal curvatures, which result in loss of vision, to become mainly irregular astigmatism and myopia, and the secondary causes are sometimes corneal scarring [1, 2]. It is known that keratoconus is caused by various factors, which include biomechanical changes of the cornea and inflammatory and genetic factors; however, not all of them have been identified yet [3–5].

The clinical findings of keratoconus evaluated with slit-lamp biomicroscopy and retinoscopy are likely to be various according to the progression and severity of the disease [6]. Although clinical findings also play a crucial role in keratoconus diagnosis, corneal topography is currently considered the primary diagnostic tool [7]. The slit-lamp examination or refraction test can easily diagnose advanced stage keratoconus rather than the early stage keratoconus; therefore, the corneal topography is the diagnostic tool for determining the changes in the early corneal stage of keratoconus. In addition, abnormal posterior elevation, irregular corneal thickness distribution, and the differentiation of other inflammatory corneal diseases that cause corneal deformity are critical for keratoconus diagnosis. However, various corneal topography modalities and variations in the reference values for each topographic map have been used for keratoconus diagnosis [8, 9]. Therefore, early diagnosis approaches for keratoconus using various corneal topography have been proposed, and one of them is the corneal elevation map method [10, 11]. Elevation-based topography (tomography) systems are currently the most broadly used systems for diagnosing keratoconus [12]. However, the main disadvantage of topographical modalities is their sensitivity to a poor ocular surface, such as unstable tear film and corneal scars.

Furthermore, the swept-source optical coherence tomography (SS-OCT), which has a higher speed and good sensitivity, has been used to measure the anterior and posterior corneal topographies, as well as the cross-sectional corneal tomographic images [13, 14]. Notably, it has been proven to be precise and reproducible in measuring cross-sectional pachymetry with wide corneal coverage and can differentiate patients with clinical keratoconus from normal patients [15].

The novel SS-OCT (ANTERION) is an accurate and highly reproducible method for evaluating the anterior segment [16–18]. Previous studies have shown the effectiveness of the SS-OCT for eye measurements such as corneal curvature, anterior angle, and axial length [19, 20]. SS-OCT generates a corneal topographic map based on an elevation map; however, no study has been conducted on the usefulness of the elevation map based on the optimal reference surface specifically for keratoconus diagnosis. Therefore, this study aimed to evaluate the usefulness of keratoconus diagnosis using elevation maps generated from the SS-OCT ANTERION.

## Materials and methods

We retrospectively reviewed the medical records of patients who underwent SS-OCT at Kim’s Eye Hospital from February 2020 to February 2021 to evaluate the anterior segment. The study protocol was approved by the Institutional Review Board (IRB number: 2021-12-009) of Kim’s Eye Hospital, Seoul, Korea. In addition, the study was conducted following the protocols of the Declaration of Helsinki.

Overall, 43, 37, and 46 eyes of patients with keratoconus, keratoconus suspect, and normal corneas were included in this study. Keratoconus suspect and keratoconus diagnoses were based on the clinical slit-lamp findings and characteristic patterns based on Scheimpflug–Placido topography (SIRIUS^®^, Costruzione Strumenti Oftalmici, Florence, Italy). Group 1: One eye from each patient with keratoconus was included. Keratoconus was diagnosed when one or more of the following clinical outcomes were observed: anterior bulging of the cornea, stromal thinning, Fleischer ring, or Vogt striae on the slit-lamp examination and asymmetric bow-tie pattern with or without skewed axes, and central or paracentral steepening of the cornea on topographic findings. Eyes with grade I, II, and III keratoconus based on the Amsler–Krumeich classification (keratometric astigmatism < 10.00 diopters [D], mean central K reading > 53.00 D, absence of corneal scarring, or minimum corneal thickness > 300 μm), and no treatment history for keratoconus were included. Since corneal opacity or hydrops may cause errors in the corneal topography measurement, corneal scarring was excluded. Group 2: Suspected keratoconus eyes were defined as a case of normal corneal outcomes on a slit-lamp biomicroscopy and abnormal localized steepening or asymmetric bow-tie pattern on the corneal topography, and one or more of the following findings: keratometric power > 47.0 D, oblique cylinder > 1.50 D, central corneal thickness < 500 μm, and clinical keratoconus in patient’s eyes [7]. Group 3: Eyes were considered normal if they had no ocular pathology, no previous ocular surgery, no significant refractive error, and no irregular corneal pattern on the corneal topography. The exclusion criteria in all groups were glaucoma, suspicion of glaucoma, intraocular pressure-lowering medications, corneal scarring, severe dry eye, pregnancy or nursing, current corneal infection, or an underlying autoimmune disease. In addition, only one eye was randomly selected in all patients.

Furthermore, all patients’ eyes underwent a complete ophthalmologic examination, including a best- corrected visual acuity test, non-contact tonometer, and mydriatic fundus examination. Before the SS-OCT test, patients were instructed to fix their heads accurately and blink their eyes so that tears were uniformly applied to the corneal surface. The examination was performed in cataract mode by a skilled operator. The measurement results confirmed that all three groups that correspond to the acquisition quality parameters (“Motion,” “Fixation,” and “Tear film and lid”) were all “pass,” and if any pass failed to appear, the examination was repeated.

### Instrument

ANTERION (Heidelberg Engineering Inc., Heidelberg, Germany) is a novel SS-OCT device that captures a broader scan depth (14.5 mm) and width (16.5 mm) using a light source of 1300-nm wavelength, with high axial and lateral resolutions of < 10 μm and < 35 μm, respectively, and a scan speed of 50.000 A-scan/second.

The corneal curvature was measured using the SS-OCT images alone (total of 65 radial B-scan images, 256 A-scans per B-scan) with 9 mm length. The corneal maps generated from the SS-OCT image data were 8 mm in diameter [21]. Study parameters included the simulated anterior axial curvature (Sim-K), posterior axial curvature, and ray-traced total corneal power in the 3-mm zone of the central cornea; central corneal thickness; and thinnest corneal thickness. A fit zone diameter of 8 mm was applied to the instruments. The anterior and posterior elevations at the thinnest point were compared in the normal, keratoconus suspect, and keratoconus eyes based on the best-fit sphere (BFS) and toric-ellipsoid (BFT).

### Statistical analysis

The data were analyzed using the Statistical Package for Social Sciences (SPSS) Statistics for Windows (version 22.0; IBM Corp., Armonk, NY, USA). The Shapiro–Wilk test was used to evaluate the normality of the numerical data. The baseline characteristics and anterior segment parameters among the three groups were compared using the Kruskal–Wallis test. The Mann–Whitney tests and Bonferroni’s adjustment were used to compare the groups. Sex differences and laterality comparisons among the groups were performed using the chi-squared test. Receiver operating characteristic (ROC) curves were designed to determine the overall predictive accuracy of each parameter, as shown by the area under the curve (AUC). These curves were obtained by plotting sensitivity versus 1—specificity, which was calculated for each observed value. An area of 100% implied that the test perfectly differentiated the groups. We also employed this approach to identify the cut-off points for the studied parameters to maximize the sensitivity and specificity (Youden’s index) in differentiating between keratoconus and suspected keratoconus cases from normal corneas. The following statistical metrics were used to evaluate the performances of the eligible patient cut-off point: positive likelihood ratio (PLR > 10 indicates convincing diagnostic evidence; 5 < PLR < 10 indicates strong diagnostic evidence), negative likelihood ratio (NLR < 0.10 indicates convincing diagnostic evidence; 0.2 < NLR < 0.1 indicates strong diagnostic evidence). Statistical significance was set at *P* < 0.05.

## Results

Data for 43, 37, and 46 patients with keratoconus, suspected keratoconus, and normal corneas of the unilateral eye from a total of 126 patients were analyzed in this study. There were no statistically significant differences between the three groups (*P* > 0.05) and in the demographic data, except for the cylinder power (*P* < 0.001) (Table 1).

**Table 1:** Patient demographics of three groups.

Parameters	Normal (n = 46)	KC suspect (n = 37)	KC (n = 43)	P value
**Age (years)**	29.76 ± 10.26	28.81 ± 10.94	30.95 ± 9.11	0.153*
**Sex**				
**Male:Female (n)**	22:24	20:17	20:23	0.776^†^
**Laterality**				
**Right**	21 (45.65%)	20 (54.05%)	22 (51.16%)	0.736^†^
**Left**	25 (54.35%)	17 (45.95%)	21 (48.84%)	
**Refractive errors (D)**				
**Spherical**	-3.28 ± 2.41	-3.21 ± 2.77	-2.35 ± 2.75	0.197*
**Cylindrical**	-0.94 ± 0.85	-2.09 ± 1.86	-2.55 ± 1.98	<0.001*

KC, keratoconus; D, diopter

* Kruskal-Wallis test. P < 0.05 is statistically significant.

^†^ Chi-square teat. P < 0.05 is statistically significant.

Table 2 presents the mean ± standard deviation values of the anterior, posterior, and total keratometric values and positions, central and thinnest pachymetry, and anterior and posterior elevations in the three groups. All keratometric parameters were significantly different between the three groups, except for Kmax X and Y position values (Kruskal–Wallis test, *P* < 0.05). In the post-hoc test, a significant difference was not observed only in the posterior astigmatism value between the normal group and the keratoconus suspect group. However, the central and thinnest corneal thicknesses significantly differed among the three groups (Kruskal–Wallis test, *P* < 0.001). Statistically significant differences between each group comparison in the post hoc test were observed except between the normal and keratoconus suspect groups. In addition, the thinnest corneal position showed a significant difference along the Y-axis.

**Table 2:** Anterior segment parameters of ANTERION in three groups.

Parameters	Normal (n = 46)	KC suspect (n = 37)	KC (n = 43)	P value*	P value^†^	P value^‡^	P value^§^
**Mean ant Sim K (D)**	43.29 ± 1.73	44.64 ± 1.29	48.12 ± 6.22	<0.001	<0.001	<0.001	<0.001
**Steep (D)**	44.13 ± 1.96	45.91 ± 1.77	50.04 ± 7.27	<0.001	<0.001	<0.001	<0.001
**Flat (D)**	42.50 ± 1.66	43.47 ± 1.26	46.40 ± 5.61	<0.001	0.005	<0.001	<0.001
**ant astigmatism (D)**	1.62 ± 1.05	2.44 ± 1.60	3.64 ± 2.92	<0.001	0.018	0.012	<0.001
**ant K max (D)**	44.71 ± 2.01	46.73 ± 1.71	54.66 ± 11.05	<0.001	<0.001	<0.001	<0.001
**X(position)**	-0.21 ± 0.71	-0.08 ± 0.70	0.04 ± 0.49	0.306	0.956	0.152	0.220
**Y(position)**	-0.69 ± 1.86	-0.75 ± 1.47	-1.17 ± 0.87	0.481	0.855	0.257	0.335
**Mean post K (D)**	-6.18 ± 0.28	-6.40 ± 0.24	-7.23 ± 1.32	<0.001	<0.001	<0.001	<0.001
**Steep (D)**	-6.41 ± 0.34	-6.66 ± 0.30	-7.64 ± 1.42	<0.001	0.001	<0.001	<0.001
**Flat (D)**	-5.97 ± 0.25	-6.16 ± 0.23	-6.97 ± 1.27	<0.001	<0.001	<0.001	<0.001
**post astigmatism (D)**	-0.44 ± 0.18	-0.50 ± 0.22	-0.76 ± 0.48	<0.001	0.341	0.002	<0.001
**post K max (D)**	-6.48 ± 0.35	-6.80 ± 0.33	-9.09 ± 2.67	<0.001	<0.001	<0.001	<0.001
**X (position)**	0.06 ±0.19	-0.03 ± 0.25	-0.02 ± 0.48	0.157	0.049	0.241	0.538
**Y (position)**	-0.40 ± 1.68	-0.42 ± 1.43	-0.91 ± 1.01	0.322	0.711	0.105	0.307
**Mean total K (D)**	42.74 ± 1.76	44.17 ± 1.38	48.06 ± 7.08	<0.001	<0.001	<0.001	<0.001
**Steep (D)**	43.49 ± 1.98	45.36 ± 1.89	49.84 ± 7.99	<0.001	<0.001	<0.001	<0.001
**Flat (D)**	42.00 ± 1.68	43.00 ± 1.25	46.28 ± 6.33	<0.001	0.003	<0.001	<0.001
**Total K (D)**	1.49 ± 1.02	2.35 ± 1.61	3.57 ± 2.71	<0.001	0.013	0.011	<0.001
**Central corneal thickness (μm)**	539.74 ± 32.07	535.95 ± 26.65	492.97 ± 49.47	<0.001	0.586	<0.001	<0.001
**Thinnest corneal thickness (μm)**	537.11 ± 31.56	532.43 ± 26.55	475.19 ± 52.31	<0.001	0.501	<0.001	<0.001
**Thinnest point X (position)**	-0.01 ± 0.40	-0.04 ± 0.49	0.06 ± 0.63	0.536	0.748	0.288	0.421
**Thinnest point Y (position)**	-0.38 ± 0.23	-0.42 ± 0.31	-0.71 ± 0.47	<0.001	0.728	<0.001	<0.001
**Anterior elevation (μm), BFS**	2.53 ± 1.44	5.04 ± 2.17	22.65 ± 18.56	<0.001	<0.001	<0.001	<0.001
**Anterior elevation (μm), BFT**	2.57 ± 1.23	5.72 ± 2.08	24.36 ± 19.68	<0.001	<0.001	<0.001	<0.001
**Posterior elevation (μm), BFS**	5.51 ± 4.24	10.35 ± 7.72	57.01 ± 43.88	<0.001	<0.001	<0.001	<0.001
**Posterior elevation (μm), BFT**	5.66 ± 3.44	11.60 ± 7.71	60.93 ± 47.39	<0.001	<0.001	<0.001	<0.001

KC, keratoconus; D, diopter; BFS, best-fit sphere; BFT, best-fit toric-ellipsoid

* Kruskal-Wallis test. P < 0.05 is statistically significant.

^†^ Mann-Whitney U test with Bonferroni correction between the normal and suspected KC groups. P < 0.05 is statistically significant.

^‡^ Mann-Whitney U test with Bonferroni correction between the normal and KC groups. P < 0.05 is statistically significant.

^§^ Mann-Whitney U test with Bonferroni correction between the KC suspect and KC groups. P < 0.05 is statistically significant.

**Fig 1:**
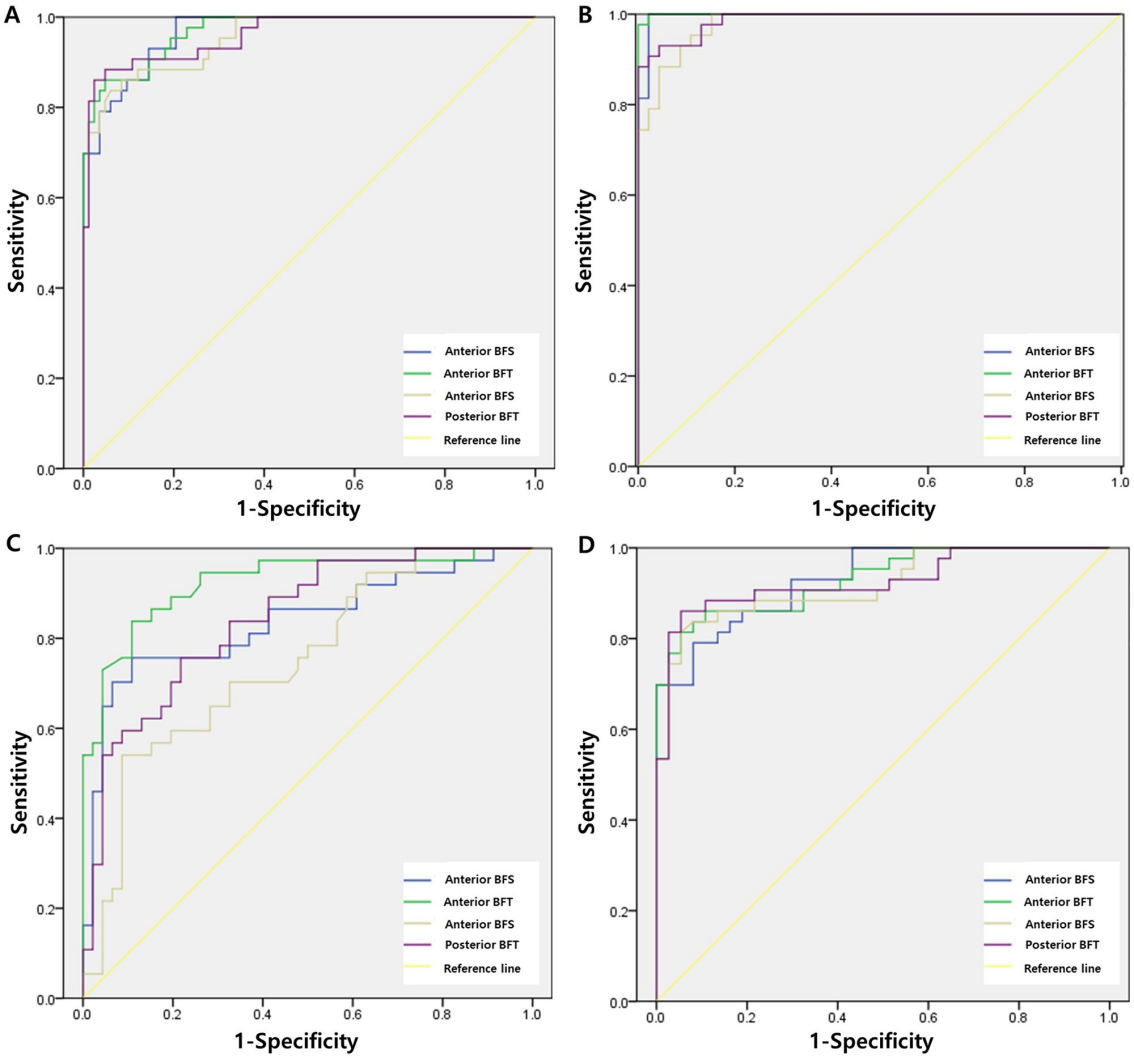
Receiver operator characteristic (ROC) curves analysis for keratoconus versus keratoconus suspect or normal cornea. (A) ROC curves for keratoconus versus normal and keratoconus suspect cornea (B) ROC curves for normal versus keratoconus cornea (C) ROC curves for keratoconus suspect versus normal cornea (D) ROC curves for keratoconus versus keratoconus suspect cornea.

Furthermore, there were statistically significant differences in anterior and posterior elevation values obtained with all reference surfaces in the keratoconus group compared with the normal and keratoconus suspect groups (Kruskal–Wallis test, *P* < 0.001 and post hoc test, *P* < 0.001). Fig 1 shows the comparison results of the ROC curve analyses for keratoconus and keratoconus suspect with those for normal corneas. In the keratoconus group, the area under the ROC curve for the normal group and the keratoconus suspect group was 0.967 and 0.969 and 0.953 and 0.961 for the anterior BFS and BFT and posterior BFS and BFT, respectively. The independent ROC values for the normal corneal group and the keratoconus group were 0.987 (versus normal group) and 0.920 (versus keratoconus suspect group) for anterior BFS, 1.000 (versus normal group) and 0.926 (versus keratoconus suspect group) for anterior BFT, 0.979 (versus normal group) and 0.917 (versus keratoconus suspect group) for posterior BFS, and 0.991 (versus normal group) and 0.932 (versus keratoconus suspect group) for posterior BFT. In addition, the highest ROC value was observed when the BFT reference surface was used to diagnose keratoconus. Table 3 shows the results of the ROC curve analysis, best cut-off point, and sensitivity and specificity of the best cut-off points for each parameter tested in the keratoconus group versus the normal eyes and keratoconus suspect groups. In diagnosing the keratoconus group compared to the normal and keratoconus suspect groups, the sensitivity was 93% and specificity of 85% with an anterior BFS of 5.87 μm, the sensitivity of 85%, and specificity of 85% with an anterior BFT of 8.05 μm, the sensitivity of 83%, and specificity of 94% with a posterior BFS of 14.92 μm, the sensitivity of 85%, and specificity of 98% with a posterior BFS of 16.44 μm. Furthermore, relatively higher sensitivity and specificity were observed when the BFT reference surface was used than when the BFS reference surface was used in the study.

**Table 3:** The cut-off point, specificity, and sensitivity values identified by the different reference surface.

	Thinnest point	AUROC	SE	P	Cut-off (μm)	Se (%)	Sp (%)	PLR	NLR
**KC vs KC suspect + normal**	**Ant. BFS**	0.967	0.013	0.000	4.97	100	80	4.88	0
	**Ant. BFT**	0.969	0.013	0.000	8.83	84	96	23.16	0.17
	**Post. BFS**	0.953	0.017	0.000	14.92	84	94	13.90	0.17
	**Post. BFT**	0.961	0.017	0.000	16.44	86	98	35.71	0.14
**KC suspect vs Normal**	**Ant. BFS**	0.836	0.047	0.000	3.73	76	89	6.96	0.27
	**Ant. BFT**	0.925	0.031	0.000	4.03	81	89	7.46	0.21
	**Post. BFS**	0.749	0.054	0.000	9.68	54	88	6.22	0.50
	**Post. BFT**	0.841	0.043	0.000	10.14	60	91	6.84	0.44
**KC vs Normal**	**Ant. BFS**	0.996	0.004	0.000	4.95	100	98	46.00	0
	**Ant. BFT**	0.999	0.001	0.000	5.06	98	100	n.a.	0.02
	**Post. BFS**	0.981	0.010	0.000	9.88	95	89	8.77	0.05
	**Post. BFT**	0.988	0.007	0.000	11.13	93	96	21.40	0.07
**KC suspect vs KC**	**Ant. BFS**	0.930	0.026	0.000	7.76	79	92	9.75	0.23
	**Ant. BFT**	0.931	0.027	0.000	8.96	81	95	15.06	0.20
	**Post. BFS**	0.919	0.031	0.000	15.64	81	95	15.06	0.20
	**Post. BFT**	0.926	0.030	0.000	16.44	86	95	15.92	0.15

AUROC, area under the receiver operating characteristic curve; CI, confidence interval; KC, keratoconus; BFS, best-fit sphere; BFT, best-fit toric-ellipsoid; SE, standard error; Se, sensitivity; Sp, specificity; PLR, positive likelihood ratio; NLR, negative likelihood ratio; n.a, not applicable

## Discussion

The new SS-OCT ANTERION was used in this study to assess multiple corneal parameters, including anterior, posterior, and total keratometric values, central and thinnest corneal thicknesses, and elevation at the thinnest point. These parameters were compared with normal control, keratoconus suspect, and keratoconus. The keratoconus group had considerably greater mean keratometric and corneal elevation values than the other groups. Notably, this difference in corneal elevation was more evident in the BFT reference surface.

HB Fam et al. revealed that the anterior corneal elevation parameters are clinically appropriate measures for diagnosing keratoconus and keratoconus suspect eyes [22]. Previous studies reported that anterior and posterior elevations were the most effective parameters for the keratoconus diagnosis [23, 24]. Table 4 shows the previously studied results compared with other corneal topography since no study has examined corneal elevation cut-off value as a reference value for keratoconus diagnosing using the new SS-OCT ANTERION. Previous studies using various devices, corneal elevation, and cut-off values indicated excellent sensitivity and specificity for diagnosing keratoconus. However, it can be observed that there is a discrepancy in the absolute values of the measured parameters using different equipment and study population. It is believed that the discrepancies in the results are due to the difference in the patient group, measurement method and principle, and reference surface. Therefore, information on cut-off values applicable to each device is important in diagnosing keratoconus. This study helps diagnose patients with keratoconus using the new SS-OCT ANTERION by showing the anterior segment parameters and cut-off values of corneal elevation values.

**Table 4:** Cut-off point, specificity, and sensitivity values identified in previous study.

Study	Device	Reference surface	Elevation values(μm)	Normal control	FFKC or KC suspect	KC	Cut-off value (μm)	Sensitivity	Specificity
**M Itoi et al**. [38] **(Normal n = 88, FFKC n = 13, KC n = 29)**	SS-OCT CASIA 1000	BFS (central 5 mm of the anterior or posterior cornea)	anterior	5.43 ± 1.38 (n = 88)	13.85 ± 7.23 (n = 13)	44.00 ± 22.15 (n = 29)	8.00	85%	96%
			posterior	10.00 ± 2.82	22.69 ± 12.07	89.93 ± 41.93	13.00	85%	86%
**Rao et al**. [39] **(Normal n = 50, KC suspect n = 60, KC n = 15)**	Orbscan II	BFS	anterior	0.005 ± 0.002	0.009 ± 0.007	0.013 ± 0.010	-	-	-
			posterior	0.021 ± 0.006	0.035 ± 0.015	0.044 ±0.025	40	-	-
**I Kovács et al**. [23] **(Normal n = 41, KC n = 41)**	Pentacam	BFS (central 5 mm of the anterior or posterior cornea)	anterior	2.7 ± 2.3	-	33.3 ± 28.6	-	-	-
			posterior	5.7 ± 5.5		55.8 ± 33.2	15.5	95.1%	94.3%
**I Kovács et al**. [29] **(Normal n = 70, KC n = 44)**	Pentacam	BFS, BFTE (fixed 8-mm-diameter)	Posterior elevation BFS 8 mm	1.38 ± 5.75	-	42.68 ± 33.44	15.5	91%	98%
			Posterior elevation BFTE 8 mm	5.66 ± 5.02		67.57 ± 48.57	10.5	91%	95%
**Orucoglu F et al**. [40] **(normal n = 513, KC n = 656)**	Pentacam	BFS (8-mm-diameter)	Anterior	3.59 ± 2.43	-	16.28 ± 12.14	8.5	71.6%	100%
		at thinnest point	Anterior elevation at thinnest point	2.29 ± 1.80		20.53 ± 14.33	5.5	91.3%	97.4%
		BFS (8-mm-diameter)	Posterior	5.64 ± 3.50		35.74 ± 25.95	12.5	87.3%	96.1%
		at thinnest point	Posterior elevation at thinnest point	6.41 ± 3.83		47.39 ± 28.52	13.5	93.2%	94.9%
**Mostafa EM et al**. [41] **(Normal n = 500, KC n = 100)**	Sirius	Elevation values at Thinnest corneal point	Spherical MAE	5.2 ± 1.9	-	21.2 ± 11.3	24	91%	92%
			Aspherotoric MAE	5.6 ± 2.4		23.9 ± 17.1	19	98%	99%
			Spherical MPE	6.3 ± 2.0		28.9 ± 18.1	15	92%	93%
			Aspherotoric MPE	6.7 ± 2.3		29.8 ± 17	12	98%	99%
**Smadja D et al**. [42] **(Normal n = 177 FFKC n = 47 KC n = 167)**	GALILEI	BFS and BFTA (8-mm-diameter)	BFS MAE	5.4 ± 3.15	7.2 ± 4.3	25.2 ± 12.5	11	92%	91%
			BFTA MAE	4.8 ± 1.7	8.4 ± 3.9	31.9 ± 15.8	9	98%	99%
			BFS MPE	13.1 ± 5.2	15.4 ± 6.5	46.0 ± 20.6	21	93%	95%
			BFTA MPE	8.6 ± 2.8	16.9 ± 6.9	57.8 ± 28.4	16	99%	99%

FFKC, Forme-Fruste Keratoconus; KC, keratoconus; BFS, best-fit sphere; BFTA, best-fit toric and aspherical surface; BFTE, best-fit toric-ellipsoid; MAE, mean anterior elevation; MPE, mean posterior elevation

The area under ROC (AUROC) curve is a plot of sensitivity against 1 − specificity, which implies true versus false positives. This area ranges from 1 (100%) to 0.5 (50%), which represents perfect discrimination and discrimination being no better than chance. Furthermore, between that range, 0.90–1 represents excellent discrimination, 0.80–0.90 good, 0.70–0.80 fair, 0.60–0.70 poor, and 0.50–0.60 very poor [25]. An area of 0.5 represents a completely inefficient measure. Therefore, in this study, the anterior and posterior elevation values with BFS and BFT reference surfaces at the thinnest point measured using the SS-OCT ANTERION showed that AUROC ranges from 0.749–0.925 for discriminating normal eyes from that of the keratoconus suspect, and 0.9 or higher in all other cases, indicating clinically useful data for differentiating normal eyes from keratoconus.

This study obtained better AUROC results when the BFT reference surface was used rather than the BSF reference surface. The cornea is aspherical and has different toricities on the anterior and posterior surfaces. Due to these morphological and different biomechanical characteristics of the anterior and posterior portions of the cornea, different changes occur in the anterior and posterior surfaces of patients with keratoconus. However, corneal elevation can be analyzed relative to the reference surface. Therefore, a reference surface that is both toric and aspherical would correspond better with the real corneal shape, and it might be helpful to detect local corneal changes and abnormalities more sensitively [26]. It is established that posterior corneal elevation in keratoconus is steeper than the anterior elevation [27, 28]. Posterior corneal elevation can effectively discriminate keratoconus from normal corneas, although the measured values and cut-off points depend on selecting reference surface and corneal asphericity. In addition, a previous study suggests that the toric ellipsoid reference surface is the most sensitive method for differentiating keratoconus [29]. Notably, corneal asphericity correlated with keratometric and pachymetric results, in which the parameters were characteristic indicators of keratoconus progression. Furthermore, toric ellipsoid reference surfaces approximate the aspheric corneal surface better than the spherical models for the early diagnosis of keratoconus [30, 31].

Although the thinnest point of corneal thickness and that of the corneal elevation keratoconus are not completely overlapped [32], “tomographic” observations such as corneal elevation and progressive corneal thinning are important criteria for the initial diagnosis and judging of keratoconus progression [12]. In a recent study, anterior and posterior corneal parameters, which include the corneal thickness of keratoconus using SS-OCT, showed better results in repeatability and reliability than the existing Scheimpflug–Placido topography technique [33, 34]. It is assumed that this difference is due to the higher number of radial scans (65 images of SS-OCT versus 25 images in the Scheimpflug–Placido topography), shorter scan times (< 1 sec vs. 1–2 sec in Scheimpflug–Placido topography), and the real-time eye tracking system of SS-OCT. Therefore, it is believed that SS-OCT is advantageous for measuring tomographic changes such as corneal thickness and elevation in keratoconus.

This study had some limitations. First, it had a retrospective design and a smaller sample size. Therefore, there might have been a selection bias in retrospectively differentiating the keratoconus group from the normal eye group. In addition, there were no findings on the difference in corneal elevation values according to keratoconus grade due to the small number of study participants. It is suggested that additional studies are required for each keratoconus grade with more study participants in the future. Second, the participants in the study were Asian. Consequently, our results may be inapplicable to other ethnicities. However, the incidence rate in the general population was known to be 5.56 cases (95% confidence interval (CI): 5.47–5.66) per 100,000 person-years in South Korea [35]. A previous study [36] found that Asians had a higher incidence of keratoconus than Caucasians, and a recent epidemiologic study using Pentacam on Caucasians reported that the prevalence was 1.2% [37]. Therefore, this difference in incidence is believed to affect the difference in severity at diagnosis. Future studies using SS-OCT in more participants and various races are required accordingly. In addition, only the SS-OCT single equipment was evaluated and analyzed in keratoconus diagnosis. Therefore, a future comparative analysis with equipment, such as other SS-OCT or Scheimpflug–Placido topography, is warranted.

In summary, this is the first study to evaluate corneal elevation associated with the anterior segment measurements and diagnosis of patients with keratoconus using a new type of SS-OCT. SS-OCT is believed to have an advantage over the existing Scheimpflug device in evaluating corneal elevation. In addition, the BFT reference surface exhibited a more sensitive diagnostic power than the BFS reference surface for evaluating the anterior and posterior corneal elevations using the SS-OCT. Therefore, the reference value presented in this study can be re-evaluated through comparison with other corneal topographies in the future.

## Supporting information

S1 File Data analyzed. (XLSX)
